# Global histone modification fingerprinting in human cells using epigenetic reverse phase protein array

**DOI:** 10.1038/cddiscovery.2016.77

**Published:** 2017-03-06

**Authors:** Marina Partolina, Hazel C Thoms, Kenneth G MacLeod, Giovanny Rodriguez-Blanco, Matthew N Clarke, Anuroop V Venkatasubramani, Rima Beesoo, Vladimir Larionov, Vidushi S Neergheen-Bhujun, Bryan Serrels, Hiroshi Kimura, Neil O Carragher, Alexander Kagansky

**Affiliations:** 1 Synthetic Epigenetics Laboratory, MRC Human Genetics Unit, Institute of Genetics and Molecular Medicine, University of Edinburgh, Edinburgh, UK; 2 Edinburgh Cancer Research Centre, Institute of Genetics and Molecular Medicine, University of Edinburgh, Edinburgh, UK; 3 Department of Biosciences, University of Helsinki, PO Box 65 (Viikinkaari 1), 00014, Helsinki, Finland; 4 Department of Health Sciences and ANDI Centre of Excellence for Biomedical and Biomaterials Research, Faculty of Science, University of Mauritius, Réduit, Republic of Mauritius; 5 Developmental Therapeutics Branch, National Cancer Institute, Bethesda, MD 20892, USA; 6 Cell Biology Unit, Institute of Innovative Research, Tokyo Institute of Technology. 4259, Nagatsuta, Midori-ku, Yokohama 226-8501, Japan

## Abstract

The balance between acetylation and deacetylation of histone proteins plays a critical role in the regulation of genomic functions. Aberrations in global levels of histone modifications are linked to carcinogenesis and are currently the focus of intense scrutiny and translational research investments to develop new therapies, which can modify complex disease pathophysiology through epigenetic control. However, despite significant progress in our understanding of the molecular mechanisms of epigenetic machinery in various genomic contexts and cell types, the links between epigenetic modifications and cellular phenotypes are far from being clear. For example, enzymes controlling histone modifications utilize key cellular metabolites associated with intra- and extracellular feedback loops, adding a further layer of complexity to this process. Meanwhile, it has become increasingly evident that new assay technologies which provide robust and precise measurement of global histone modifications are required, for at least two pressing reasons: firstly, many approved drugs are known to influence histone modifications and new cancer therapies are increasingly being developed towards targeting histone deacetylases (HDACs) and other epigenetic readers and writers. Therefore, robust assays for fingerprinting the global effects of such drugs on preclinical cell, organoid and *in vivo* models is required; and secondly, robust histone-fingerprinting assays applicable to patient samples may afford the development of next-generation diagnostic and prognostic tools. In our study, we have used a panel of monoclonal antibodies to determine the relative changes in the global abundance of post-translational modifications on histones purified from cancer cell lines treated with HDAC inhibitors using a novel technique, called epigenetic reverse phase protein array. We observed a robust increase in acetylation levels within 2–24 h after inhibition of HDACs in different cancer cell lines. Moreover, when these cells were treated with *N*-acetylated amino acids in addition to HDACs, we detected a further increase in histone acetylation, demonstrating that these molecules could be utilized as donors of the acetyl moiety for protein acetylation. Consequently, this study not only offers a novel assay for diagnostics and drug screening but also warrants further research of the novel class of inexpensive, non-toxic natural compounds that could potentiate the effects of HDAC inhibitors and is therefore of interest for cancer therapeutics.

## Introduction

Post-translational modifications of proteins are one of the major determinants of genomic functions, allowing a swift, controlled and reversible response to environmental signals.^1^ Such changes may be induced by phosphorylation, nitrosylation, methylation, ubiquitination and sumoylation, but arguably the most studied of all the modifications is acetylation.^2^ Addition of acetyl groups to lysine residues within a protein alters the charge and structure of the molecule, leading to a change in its function.^3^ In the past two decades, particular interest has been given to the acetylation of histone molecules, which have been shown to be involved in complex combinatorial regulation of local genome properties such as transcription, DNA repair and other chromosome transactions.^4^ Histones are abundant cellular proteins required for packaging the DNA into nucleosomes and, as they contain proportionally more lysine residues than other proteins, they account for a high number of these residues within the total protein pool. When lysine residues are acetylated, the positive charge of the histone molecule is neutralized and the DNA becomes less tightly associated with the nucleosome, allowing the transcriptional machinery unhindered access to the transcription factor binding sites.^5^ Furthermore, acetylation also influences the interaction of histones with other proteins, as specialized binding domains (e.g. bromodomain) can specifically distinguish between acetylated and non-acetylated forms of the protein.^6^

Over the last decade, massive government and commercial investment into global projects, including ENCODE, Epigenome, and Epigenesys, led to the refinement of methods related to ChIP-on-chip, ChIP-seq and the introduction of new combined methods, such as ChIA-PET.^7^ The resultant mapping of a number of histone acetylations, methylations and phosphorylations onto model genomes produced overwhelmingly detailed databases that shed light on the potential genomic contexts related to the functional and structural make-up of the genome in health and disease. Despite an ongoing debate concerning the substantiation of the original histone code hypothesis for cell fate,^8^ and the difficulty in assaying causal links between locus-specific changes in histone marks and pathological chromatin changes in cancer and other diseases, the analysis of global histone modifications linked to intracellular pH and carbon metabolism represent a compelling, yet under-investigated task. It has been clearly demonstrated that the patterns of global H3 and H4 acetylations correlate with the severity of prostate and other cancers,^9^ while global methylation patterns may have a diagnostic and prognostic potential for a range of different tumours.^10^

Histone acetylation and deacetylation is a well-studied process involving the turnover of the acetyl CoA moiety by specialist enzymes, namely histone/lysine acetyltransferases (HATs/KATs) and histone deacetylases (HDACs), which contribute differentially in specific tissues and genomic contexts.^11,12^ It has long been established that many cancers are characterized by global hypoacetylation of histones.^13^ Overexpression of individual HDACs is associated with reduced survival rates in numerous tumours, including cancers of the colon, breast, lung and prostate.^14^ Furthermore, genetic knockdown of specific HDACs within these tumours induced cell cycle arrest and apoptosis. As a consequence, numerous HDAC inhibitors have undergone clinical trials and several have recently been approved by the FDA for use in various human cancers.^15^ It is hoped that these HDAC inhibitors can be used in tumours that have either developed resistance to conventional therapies or following relapse of the primary cancer.^16^ It has been clearly demonstrated that the patterns of global H3 and H4 acetylations correlate with the severity of prostate and other cancers,^9^ while global methylation patterns may have a diagnostic and prognostic potential for a range of different tumours.^10^

Furthermore, the global levels of a number of histone modifications have been independently proven to correlate with diagnosis and prognosis in lung, kidney, breast, ovary and pancreatic cancers.^17^ Therefore, the development of robust, high-throughput assays capable of accurately fingerprinting a subset of specific histone modifications within preclinical disease models or a patient biopsy warrants advancement in order to support the development of new epigenetic modifying therapies and to appropriately inform clinical decisions on the choice and course of such therapy.

Currently, the methodologies most frequently employed to investigate histone modifications are western blot analysis and immunofluorescent microscopy. However, these techniques do not lend themselves to efficient identification of histone marks, as they suffer from chromatin preparation challenges and an inaccessibility of target epitopes respectively. Furthermore, they are not readily amenable to either standardization or quantitation, and neither protocol can be efficiently scaled-up to yield high-throughput analysis of preclinical and clinical samples.

Advances in the analysis of post-translational modifications of histones and other molecules are promised by the ongoing boom in mass spectrometry. Continuing mass spectrometry studies will support the discovery of new functionally significant histone and other post-translational marks to add to current list of cancer biomarkers. Mass spectrometry studies also promises to elucidate chemical co-dependencies of epigenetic marks with metabolomics^18,19^ to unveil comprehensive mechanisms of biochemical programming at and beyond cellular and tissue homoeostasis levels. However, despite being the proteomics champion at the forefront of new discoveries, mass spectrometry is a complex and resource extensive methodology unsuitable for routine screening applications in preclinical drug discovery or clinical diagnostic settings. Therefore, there is a pressing need for the development of other proteomic methods to quantitatively fingerprint global histone acetylation and methylations levels in preclinical and patient samples at scale.

### Epigenetic reverse phase protein array (eRPPA)

: Proteomic methods referred to as reverse phase protein array (RPPA) represents a highly efficient, sensitive and cost-effective high-throughput immunoassay,^20^ in which protein extracts are immobilized on the solid phase (usually nitrocellulose) and subsequently probed with the antibodies toward targets of interest. An advantage of the RPPA format over other multiplex ELISA assays is that detection antibodies are physically separated from one another resulting in no cross reactivity and unlimited multiplexing capability. In addition, the concentration and buffer conditions can be optimized for each antibody and/or analyte to ensure optimal results. In this article we describe the development of a universal RPPA method for simultaneously quantifying a panel of global histone modifications across multiple samples using automated sample printing, antibody detection and data analysis platforms. We have chosen a panel of thoroughly characterized and standardized mouse monoclonal antibodies raised against acetylations and methylations of histones H3 and H4, developed by Hiroshi Kimura.^21,22^ We have first assessed employing conventional way for preparing the cell extracts, but have not been able to obtain reproducible results using a variety of lysis/extraction buffers at any starting material amount tested (0.01–0.5 g, data not shown). We first considered isolating purified histones using specialized kits produced by Eurogentec and Active Motif, none of which satisfied us with either the course of the procedure or the output.

We incorporated the protocol previously used for histone isolation, followed by the identification of histone modifications by mass spectrometry^23^ into the eRPPA workflow described in this article. The methodology was rapid, robust and preserved the labile histone modifications intact. We present proof-of-concept data measuring increases in histone acetylations following testing of the human colorectal cancer cell line HCT116 with known HDAC inhibitors and *N*-acetylated amino acids and describe the eRPPA protocol in full.

## Results

**Figure 1:**
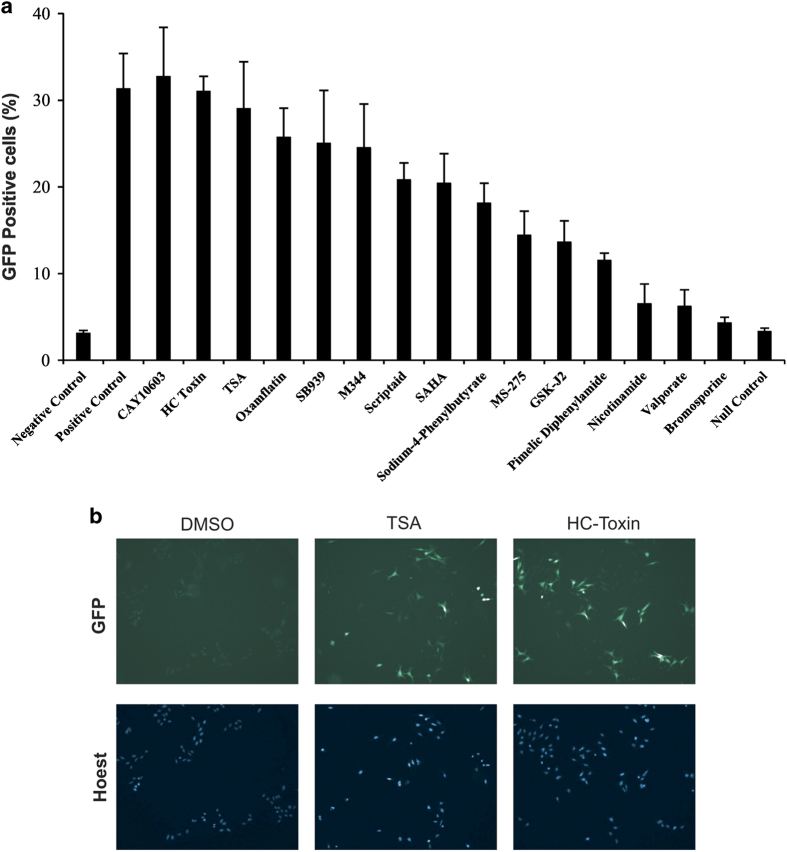
The effect of HDAC inhibitors on de-repression of GFP transgenes (**a** and **b**) HeLa-GFP cells were treated with 10 *μ*M of a panel of different HDAC inhibitors for 24 h (**a**). The percentage of GFP-positive cells (green) was then calculated as a percentage of total cells (DAPI) using fluorescent microscopy. Cells treated with vehicle alone (DMSO) served as a negative control, whereas HeLa cells transfected with an empty vector acted as null controls. TSA (10 *μ*M), which was a different batch to the one in the panel, was used as a positive control. The ‘top hits’ from the screen in (**a**) (CAY10603, HC toxin and TSA) were then used to treat HeLa-GFP cells for 24 h at a concentration of 500 nM and the effects on GFP silencing were examined by fluorescence microscopy: five biological replicates with three technical replicates each time. (**b**) The same three chemicals were then used to treat colorectal cancer cells HCT116 for 2 h at a concentration of 500 nM and the effects on acetylation of isolated histones were examined by RPPA: three biological replicates with three technical replicates each time.

The aim of this study was to develop and validate a robust method for quantifying global changes in histone acetylation from biological samples in an assay format suitable for scale-up high-throughput application across preclinical or clinical samples. To select for appropriate positive control compounds for assay development and validation, we initially screened a mini-library of known HDAC inhibitors using a human HeLa cell line with multiple integrations comprising silenced GFP transgenes.^24^ Exposure of these cells to agents that induce bulk increase of acetylation leads to de-repression of the silenced GFP transgenes, resulting in GFP expression^24^ (Figure 1a). Our screen showed that several HDAC inhibitors from our mini-library were able to de-repress silenced GFP expression (Figure 1b). Of these agents, HC toxin and CAY10603 exhibited GFP expression that was comparable to the levels observed following exposure to TSA, which served as a positive control (Figures 1a and b).

**Figure 2:**
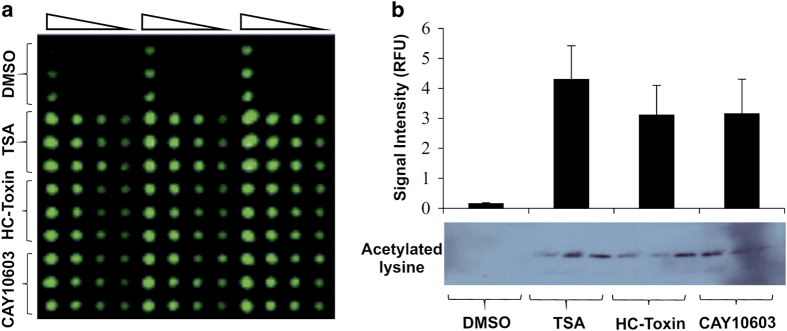
Induction of histone acetylation (**a** and **b**): Each sample, normalized for protein levels, was loaded onto the slide using twofold serial dilutions in triplicate. Panel **a** shows a typical image obtained by RPPA using a pan-acetylated lysine antibody. The amount of antibody staining was normalized with Fast Green and the staining intensity (Relative Fluorescent Units) of each sample was calculated as a percentage of cells treated with DMSO alone±S.E.M. (**b**, chart). Each value represents the mean of three individual experiments, conducted and spotted in triplicate. Ten micrograms of the isolated histone protein from each treatment was used to confirm the RPPA findings by western blot analysis using a pan-acetylated lysine antibody (**b**, below the chart); three biological replicates with three technical replicates each time.

While the eRPPA assay protocol is applicable to any cancer cell line or other cell types that can be propagated *in vitro *development, we chose HCT116 human colorectal cell line because HDACs are currently undergoing clinical trials for colorectal cancer (CRC),^25^ and HCT116 is among most commonly used human colorectal cell lines. We examined the effects of our selected drugs on histone acetylation/methylation using the new high-throughput assay devised to examine multiple histones concurrently, and we call it eRPPA. This method allows the simultaneous and quantitative analysis of histone modifications across multiple moieties, thereby enabling us to determine precisely which lysine's are altered following a treatment (Figure 2a). As expected, we found that global histone acetylation was dramatically increased following treatment with the various HDAC inhibitors (Figure 2b). While CAY10603 is HDAC6 specific, and TSA is a poor inhibitor of HDAC8, we have decided to further focus on HC toxin, since from the literature we assume it inhibits most HDACs in the human cells.

Western blots with pan-acetyl-lysine, H4K8ac and H4K12ac mouse monoclonal antibodies showed striking increase in acetylation on protein lysines, H4K8ac, and H4K12ac in drug-treated samples compared with DMSO-treated samples (data not shown).

We used two criteria to determine assay robustness and suitability for screening: (i) we checked for reproducibility across replicates and (ii) determined signal-to-noise between negative and positive controls to calculate coefficient of variance (CV) and Z′ factor, respectively.

Acetyl l-carnitine (ALCAR) was previously reported to serve as the donor of acetyl moiety for the acetylation of histones.^26^ The cleavage of the *N′*-acetyl group from amino acids is processed by the single enzyme, amino acylase, Acy1. We tested, if the observed decrease in ALCAR was due to the increased expression of Acy1, due to the HC toxin treatment, and hence, hyperacetylation of the Acy1 promoter. However our thorough analyses of the levels of Acy1 transcript (Supplementary Figure S2) and protein (not shown), in response to the treatment with HC toxin, do not support this hypothesis.

**Figure 3:**
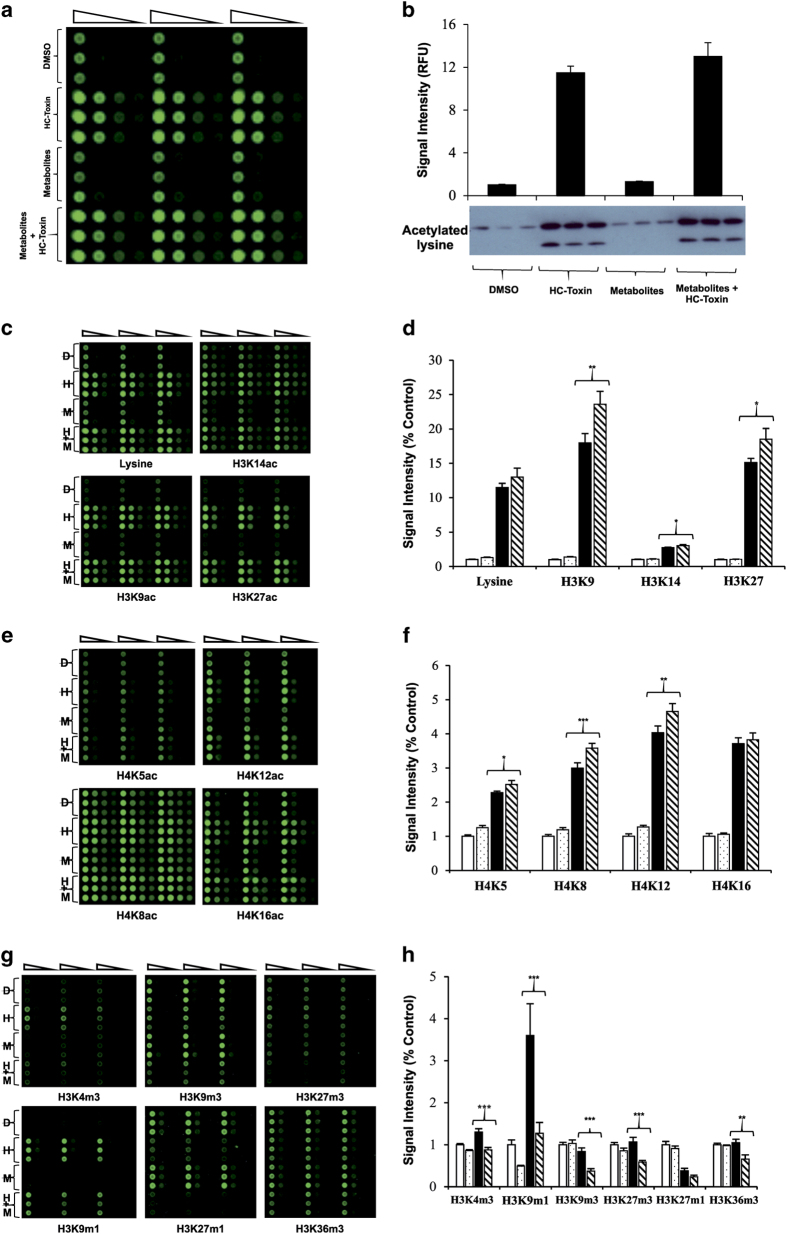
Metabolites potentiate the effects of HC toxin on histone acetylation and methylation. HCT116 cells were exposed to 100 nM HC toxin for 24 h in the presence or absence of combined metabolites (500 nM ALCAR, 500 nM NAL and 500 nM NAM) and then RPPA was conducted on isolated histones. A typical image from RPPA for pan-acetylated lysine is shown (**a**). Each sample, normalized for protein levels, was loaded onto the slide using twofold serial dilutions in triplicate. The amount of antibody staining was normalized with Fast Green and the staining intensity (Relative Fluorescent Units) of each sample was calculated as a percentage of cells treated with DMSO alone±S.E.M.; three biological replicates with three technical replicates each time. (**b**) HC toxin and HC toxin+metabolites caused a significant increase in histone acetylation, compared with DMSO or metabolites alone (*P*<0.001). The RPPA findings were confirmed using western blot analysis of the isolated histones (**b**, lower panel); three biological replicates with three technical replicates each time. Acetylation (**c**–**f**) and methylation (**g** and **h**) at specific histone residues were also examined by RPPA. Relative staining intensity was calculated and expressed as a percentage of DMSO (open bars), metabolites alone (spotted bars), HC toxin (solid bars) and HC toxin+metabolites (hatched bars). Statistical significance was determined using two-tailed Student’s *T*-test: *P<0.05; **P<0.01; ***P<0.001. Three biological replicates with three technical replicates each time for each panel (**c**–**g**).

We then determined possible synergetic effects of *N*-acetylated amino acids in combination with HC toxin with respect to histone acetylation (Figures 3a–h). The results suggest that most histone acetylations tested increase further, when HC Toxin is supplemented with the *N*′-acetylated amino acids. This result suggests that these non-toxic and inexpensive compounds have the potential to augment the therapeutic effect of HDAC inhibitors for cancer treatment, and support further research towards exploring the potential for the combination of HDAC inhibitors and acetylated amino acids in various cancers. To our knowledge, such combinatorial approaches have not been explored in clinical studies. Hypothetic potentiation of the effects of HDAC inhibitors should afford to decrease the active doses of relatively toxic HDAC inhibitors used in chemotherapy, and therefore, to reduce side effects.

## Discussion

Epigenetic alterations of histone and non-histone proteins are central events in the initiation and progression of cancer. Cancers arising from the large intestine or rectum are called CRC and represent the fourth leading cause of cancer-related death worldwide.^27^

Although general chemotherapy strategy based on fluorouracil (5-FU) is applied in CRC cases, other drugs such as irinotecan and oxaliplatin have resulted in improved outcomes,^28,29^ prompting clinicians to focus on both combination regimens in patients with metastatic disease. Interestingly, overexpression of HDAC1 in CRC correlated with significant decrease in survival and was able to predict poor patient prognosis.^30^ Further, in cases where CRCs are characterized by microsatellite instability (MSI) (approx. 15% of CRCs), which occurs due to deficiency in the mismatch repair pathway, 5-FU treatment was not effective and showed resistance as compared with microsatellite stable CRCs. This further supports our analysis of HDAC effects and the use of HCT116 cell line as the latter has a perturbed epigenetic profile and a MSI phenotype, with a strong resistance to 5-FU therapy.^31^ Therefore, analysis of histone acetylation levels would help to provide better diagnostic and prognostic strategies in the future, while HDAC inhibitors are considered as the promising route for cancer treatment. For example, HDAC inhibitor SAHA sensitized CRC tumours in model animals.^32^

We have selected HC toxin based on its potency and broad specificity against HDACs.^33^ This drug induces robust hyperacetylation of lysines of cellular proteins including histone tails in all cell lines investigated, which then stays irreversibly at a maximum level between 2 and 24 h (not shown).

In a separate set of experiments, we have examined major metabolic transitions that accompany inhibition of HDAC activity in the cells using an Orbitrap mass spectrometer (Rodriguez-Blanco and Kagansky, unpublished). Our metabolomics profiling experiments unexpectedly demonstrated statistically significant decrease in level of intracellular ALCAR, as well as other acetylated amino acids (not shown), including *N*-acetyl methionine (NAM) and *N*-acetyl aspartate (NAA). Amino acylase (Acy1) is the single mammalian enzyme that removes acetyl groups from *N′*-acetylated amino acids.^34^ Therefore, we tested if hypothetic Acy1 promoter activation, following HDAC inhibition, is responsible for the drop in the acetylated amino acids, resulted from the Acy1 overexpression. Gene expression analysis of the Acy1 transcript did not detect any overexpression of this gene caused by the HDAC inhibitor (Supplementary Figure S2). Therefore, we assume that pools of acetylated amino acids are gradually depleted as a result of the lysine hyperacetylation, as it was previously postulated for the ALCAR. We propose that other free *N*-acetylated amino acids, such as NAM and NAA, may also serve as ‘emergency’ sources of acetyl groups. Thus, it is possible that free *N*-acetylated amino acids can act as ‘acetylation sink’ to capture acetyl groups in a somewhat ‘inert’ form (by analogy to methylation sink role for 1MNA^35^), as despite the discovery of *N*-acetylated amino acids over half a century ago,^36^ very few biological roles, if any, were assigned to them in cellular metabolism.

Currently used immunohistochemistry and western blots do not provide precise means to evaluate the acetylation levels, and therefore development of novel quantitative fingerprinting approaches may serve both for the screening of patients, and for evaluating the specificity of HDAC inhibitors. eRPPA employs highly selective antibodies against histone acetylations (including key histone H3 acetylations, H3K14ac, H3K9ac, H3K27ac, and histone H4 acetylations H4K5ac, H4K8ac, H4K12ac and H4K16ac) to quantitatively detect changes in the global acetylation profiles.

The important concern with HDAC inhibitors treatment, as in the case of other types of chemotherapy, is its side effects. Although the side effects from HDAC inhibitors are comparatively mild, global changes to the protein makeover (as most of the cellular proteins are acetylated) across the whole body cannot be considered risk-free.^37^ Consequently, there will always be a ‘tug of war’ between achieving the needed HDAC inhibition on one hand and reducing the effective dose of the drug on the other.

l-Carnitine is an amino acid derivative, biosynthesized from lysine and methionine majorly in brain, kidney and liver and play important roles on lipid metabolism.^38^ ALCAR is an acetylated derivative of carnitine that has been studied and shown to act as a protective agent on many types of diseases including neurological disorders,^39^ muscle-related diseases^40^ and has very recently been shown to be a potential cancer biomarker.^41^ In the literature, it was also demonstrated that ALCAR has a potentiating effect on histone acetylation in different cancer cells. Previous study has shown that mitochondrial uptake of ALCAR can supply the cell with acetyl groups that can be utilized for histone acetylation.^26^ Other studies reported that carnitine has potentiated colon cancer cell death in Caco-2 and SW680 cells^42–44^ caused by another HDAC inhibitor, sodium butyrate. The mechanisms by which ALCAR modulates butyrate-induced cancer cell death have not been elucidated. Authors hypothesized that ALCAR may affect butyrate availability and metabolism in colon cancer cells. No change in histone H4 acetylation of butyrate-treated cells was detected in the presence of added ALCAR in the previous study.^42^ In their work, authors used western blotting with antibody against pan-acetylated H4, while acetylation of only few specific lysines on H3 and H4 was affected in our experiments. Notably in our experiments, pan-acetylated lysine antibody did not detect significant change between HC toxin-treated cell in the presence and absence of acetylated amino acids added to the media. We should also consider that butyrate is comparatively a weaker HDAC inhibitor than HC toxin.

To reveal the effects of ALCAR, NAM, and NAA on CRC cells on histone acetylation, we have used eRPPA. Despite unsurprisingly not detecting any significant effects of these compounds on their own, when combined with HC toxin, there were statistically significant increases of most acetylations tested. Interestingly, at the same time, eRPPA experiments have demonstrated substantial decrease in levels of five methylations (Figure 3h), which is likely explained by the mutually exclusive occupancy of acetyl and methyl marks on the same lysine residues. We hypothesize that increased acetylation of lysines 9 and 27 on histone H3, lysine 5 on H4 and decreased H3K9 methylation can lead to reactivation of the transcription of silenced genes in cancer, and thereby restore normal cellular growth and differentiation.

It will be important to elucidate the exact biochemical reactions involved in the transfer of acetyl groups. It is possible that amino acetyl groups cleaved from ALCAR and other acetylated amino acids by the Acy1 enzyme are incorporated into the Acetyl CoA and are then transferred to the *ε*-position on the lysines of histone tails and other proteins. Based on the current literature, we posit that ALCAR and/or NAA could act as potential therapeutic agents for CRC treatment potentiating histone acetylation in the presence of HDAC inhibitors.

It is not yet clear whether treatment of variously diagnosed CRC patients with clinically approved HDAC inhibitors in combination with *N*-acetylated amino acids could help to slow down the tumour development, stop formation of new tumours or metastases, and therefore extend the patients survival. We expect further experiments in animal CRC models and clinical trials involving non-toxic ALCAR, NAA and NAM, would answer this question.

## Materials and methods

### Chemicals

The Epigenetic Screening Library containing in excess of 140 small molecules, which target a variety of proteins, including histone deacetylases, histone acetyltransferases, methyltransferases, demethylases and acetylated histone binding proteins, were obtained from Cayman Chemicals (Ann Arbor, MI, USA). All molecules were prepared at a stock concentration of 10 mM in DMSO. Gibco Dulbecco’s modified Eagle's medium (DMEM) and fetal bovine serum (FBS) were purchased from Invitrogen (Paisley, UK). HC toxin was purchased from AbCam (Cambridge, UK). All other chemicals were supplied by Sigma-Aldrich (Dorset, UK) unless stated otherwise.

### Cell lines and drug treatment

HeLa cells containing multiple copies of GFP reporter genes, which are variously epigenetically silenced by DNA hypermethylation or the parental cell line they were derived from (null cells), were a kind gift from Prof. Richard Katz (Fox Chase Cancer Center, Philadelphia, PA, USA), and are described in detail elsewhere.^45^ HCT116, an intestinal epithelial adenocarcinoma cell line, was obtained from the European Collection of Cell Cultures (Porton Down, UK). Cells were grown as monolayers in DMEM culture medium, supplemented with 10% FBS and antibiotics (penicillin and streptomycin), and maintained under a humidified atmosphere with 5% CO_2_ at 37 °C.

For RPPA experiments with HCT116 cell line, treatments were done as follows (equivalent method was used for the RPE-1 cells (not shown)). Twelve 145 mm dishes were seeded with HCT116 cells (typically 2 million per dish). Four different treatments were done, each in three replicates (on three individual dishes), using media with added DMSO stocks of indicated drugs. Drugs were added in the indicated concentrations, with final concentration of DMSO being 0.1%. Cells were treated for 2 or 24 h in the incubator prior to collection. They were then washed with cold PBS, scraped and collected on ice in 1 ml of cold PBS. Histones were isolated using the following acid extraction protocol.

### Histone extraction

To extract histones, the culture medium was removed following treatment and the cells were scraped into ice-cold PBS and collected by centrifuging at 2000 r.p.m. The pellets were re-suspended in 1 ml of lysis buffer (250 mM sucrose, 50 mM Tris-HCl, 50 mM NaHSO_3_, 45 mM sodium butyrate, 25 mM KCl, 25 mM MgCl_2_, 10 mM *β*-mercaptoethanol, 2 mM PMSF and 0.2% Triton X-100), supplemented with 1X cOmplete Protease Inhibitor Cocktail (Sigma-Aldrich) and incubated on ice for 15 min. The nuclei were pelleted by centrifugation at 800×*g* for 10 min, the supernatant was discarded and the pellet was re-suspended in 1 ml 0.2M H_2_SO_4_. After a 30 min incubation on ice, the debris was removed by spinning at 13 000 r.p.m. for 10 min, the supernatant was transferred to a fresh tube, 125 ml of 100% TCA was added and the nuclei were incubated on ice for a further 15 min. Next, the nuclei were again pelleted by spinning at 13 000 r.p.m. for 10 min, the supernatant was discarded and the pellet was dislodged into acetone with 50 mM HCl, vortexed and incubated for 30 min at room temperature. The histones were collected by centrifuging for 5 min at maximum speed and then re-suspended in 100% acetone and left overnight at −20 °C. The tubes were then centrifuged again at 13 000 r.p.m. for 10 min, the supernatant was discarded and the histones were diluted in water with protease, phosphatase and HDAC inhibitors. The total concentration of histone proteins was determined using a Qubit Protein Assay System (Thermo Fisher Scientific, Paisley, UK). Western blotting was used to evaluate the reactivity of histone antibodies and preliminary assessment of histone. Concentrations were normalized to 100–200 *μ*g/ml and the samples were loaded on 10–20% Gradient Tricine SDS gels (Novex, Life Technologies, Carlsbad, CA, USA) for western blot analyses and Coommassie staining. Gels were run for 90 min, 125 V, according to the manufacturer’s instructions, followed by 7 min transfer to PVDF membrane using the I-Blot gel transfer system (Thermo Fisher Scientific). The membranes were hybridized with pan-acetyl-lysine, H4K8ac, and H4K12ac mouse monoclonal antibodies.

### Epigenetic reverse phase protein array

The histone samples were next subjected to RPPA Assay system (Grace Bio-Labs, Bend, OR, USA). Starting concentrations of histone samples were in the range of 400–750 *μ*g/ml. However we found that some histone modification antibodies worked well even with much lower concentration of the histones samples. Histone samples were prepared in 20% solution of glycerol in 200 *μ*l, then we added 67 *μ*l of 4× Sample Buffer (4xSB with 10% of 2-mercaptoethanol) and heated at 95 °C for 5 min. The RPPA method could accommodate higher number of samples, but to simplify our experiments we used 12 samples settings.

We loaded samples onto a 96-well V-bottom plate. The plate was divided into three areas to prepare dilutions. The first line of the wells would be 100% dilution, second – 50% dilution, third – 25% dilution, fourth – 12.5% and so on: fifth – 100%, sixth – 50%, seventh – 25%, eighth – 12.5%, etc. Sixty microlitres of 20% glycerol were added in wells in rows 2, 3, 4, 6, 7, 8 and 10, 11, 12. About 120 *μ*l of sample were added in each well of the 100% sample rows 1, 5, 9 (Supplementary Figure S1a).

Using multichannel pipette, 60 *μ*l from each of the 100% sample well was added to 50% sample well, and mixed. Sixty microlitres from 50% sample was added to 25% sample well, and mixed. Finally, 60 *μ*l of 25% sample was added to 12.5% sample well and mixed. Each plate accommodates four samples that are located in the middle of the plate in 8×16 rectangles, leaving the four-well wide border to contain only water to prevent the evaporation. Other samples were diluted the same way, adding a half of a dilution to the next well each time. After loading a sample plate, three 386-well loading plates were prepared for printing (Supplementary Figures S1b–d). When the histone samples were prepared for spotting, they were loaded into wells of sample plates as biological triplicates, 8–10 *μ*l per well. The samples were printed/spotted onto ONCYTE porous nitrocellulose film slides using Aushon BioSystems 2470 Arrayer (Billerica, MA, USA). One row contains a sample spotted in four consecutive twofold dilution series in triplicate to quantify protein in each sample. The slides were dried overnight at 4 °C and the RPPA Assay was performed using the Grace Bio-Labs protocol with a panel of histone antibodies.

Each slide/chip was processed in a hybridization cassette (Arraylt Corporation, Sunnyvale, CA, USA) to allow the detection of histone marks individually in each of 16 arrays with primary antibodies. The slide was washed with water four times for 15 min to remove salts and detergents, which could be performed in a 50 ml Falcon tube or a tray. This and all following steps should be performed with agitation. The slides were placed in an Array-It chamber. To enhance availability of an antigen-binding sites through denaturation, incubation with Antigen Retriever (Reblot strong solution; Millipore, Billerica, MA, USA) was performed for 15 min. The slides were completely covered and washed twice with water, followed by two PBS washes for 5 min each and incubated for 10 min in Superblock (Thermo). This was followed by a TBST wash, twice for 5 min and incubated with primary antibodies, at 1:250 dilutions in Superblock for 1 h. After incubation, they were again washed with TBST twice for 5 min and incubated for 10 min with blocking buffer, following which they were washed three times for 5 min using TBST. Secondary fluorescent Dylight-800-labelled anti mouse antibodies (Cell Signaling, Danvers, MA, USA) at a dilution of 1:2500 was added and incubated for 30 min in Superblock. From here on, the methodology should be carried out in dark, as the antibodies are light sensitive. Following incubation with secondary antibody, they were washed twice with TBST for 5 min, rinsed with fresh water and finally the slides were dried at room temperature for 10 min before acquiring the data. Data were collected using an Innoscan 710 IR (Innopsys, Carbonne, France) infra-red microarray scanner and the calculations were done using Mapix software (version 7.3.1; Innopsys). The calculated data are normalized in order to correct for technical variability. We used Fast Green reference staining that allowed us to account for total protein that was printed on the slide at each spot immobilized on the membrane and normalized specific antibodies signals accordingly.

### List of antibodies used in eRPPA

pan-acetyl-Lysine (AKL5C1; Santa Cruz Biotech, Santa Cruz, CA, USA); H4K5ac; H4K8ac; H4K12ac; H4K16ac; H3K9ac; H3K14ac; H3K4m1; H3K4m2; H3K4m3; H3K9m1; H3K9m2; H3K27m1; H3K27m2; H3K27m3 (all affinity purified mouse monoclonal antibodies from Kimura Lab, Yokohama, Japan).

### Fast Green staining procedure

The slide with spotted protein samples was washed with water in a 50 ml Falcon tube for 5 min with agitation. It was washed with 1% sodium hydroxide for 15 min with agitation and rinsed briefly by submerging them repeatedly 10 times in dH_2_O. Then we washed the slide in water for 10 min with agitation. The slide was placed in de-stain solution (10% acetic acid, 30% methanol in water) for 15 min with agitation. Fast green stain solution (1× in water) was added to perform the staining for 3 min with agitation. We rinsed the slide briefly with water by submerging it repeatedly in water about 10 times. We dried the slide by brief centrifugation and scanned the slide at 785 nm.

### Detection of de-repression of silenced GFP transgenes

Cells were cultured and treated in 96-well flat-bottom plates as described above. Following incubation, the medium was removed, the monolayers were washed twice with ice-cold PBS and the cells were fixed in 4% paraformaldehyde solution for 30 min. They were then washed again and stained with 30 *μ*l/well DAPI at a concentration of 1 *μ*g/ml in PBS. After 30 min exposure in the dark, the cells were washed and then visualized by fluorescent microscopy using a Zeiss Observer Z1 microscope (Zeiss FLUAR ×10/0.5) with MetaMorph v7.8.8.0 software from Molecular Devices (Sunnyvale, CA, USA). The proportion of GFP fluorescent cells was calculated as a percentage of total cells using CellProfiler v2.1.1 software.^46^ Only when a cell was deemed to be of the appropriate size, tested positive for DAPI staining and had a significantly high level of GFP staining relative to control levels, did this count as a cell with re-activated GFP reporters.

### Quantitative reverse transcriptase polymerase chain reaction (qRT-PCR)

Following treatment as described in the figure legends, HCT116 cells were scraped into ice-cold PBS and collected by centrifugation. RNA was extracted from the cells using TRIzol reagent (Ambion, Waltham, MA, USA) according to the manufacturer’s instructions and cDNA was prepared using standard procedures in a reaction containing 1 *μ*g RNA and the following reagents (all supplied by Promega, Madison, WI, USA): 50 mM Tris-HCl, 75 mM KCl, 3 mM MgCl_2_, 10 mM DTT, 1 mM nucleotides, 0.5 *μ*g Oligo(dT) primer, 100U M-MLV reverse transcriptase and 20U RNasin. Quantitative PCR was performed using SYBR Green (Applied Biosystems, Foster City, CA, USA) according to the manufacturer’s instructions with an LC480 light cycler (Roche, Basel, Switzerland). The following primers were used: ACY1 For 5′- CTTCGGGCGGGAGGCATA-3′; ACY1 Rev 5′- CCACTTTTGGCATCGAGGT-3′; The ΔCT value for each test primer compared with *Pol*II was determined and 2^ΔΔCT^ was calculated and expressed as a percentage of cells treated with vehicle alone.

## Notes

Supplementary Informatio

Supplementary Informatio

Supplementary Informatio

Supplementary Informatio

Supplementary Informatio

Supplementary Informatio

Supplementary Informatio

Supplementary Informatio

## References

[bib1] Khoury GA, Baliban RC, Floudas CA. Proteome-wide post-translational modification statistics: frequency analysis and curation of the swiss-prot database. Sci Rep 2011; 1: 90. 10.1038/srep00090 PMC3201773 22034591

[bib2] Choudhary C, Weinert BT, Nishida Y, Verdin E, Mann M. The growing landscape of lysine acetylation links metabolism and cell signalling. Nat Rev Mol Cell Biol E 2014; 15: 536–550. 10.1038/nrm3841 25053359

[bib3] Azevedo C, Saiardi A. Why always lysine? The ongoing tale of one of the most modified amino acids. Adv Biol Regul 2016; 60: 144–150. 26482291 10.1016/j.jbior.2015.09.008

[bib4] Kouzarides T. Chromatin modifications and their function. Cell 2007; 128: 693–705. 17320507 10.1016/j.cell.2007.02.005

[bib5] Bannister AJ, Kouzarides T. Regulation of chromatin by histone modifications. Cell Res 2011; 21: 381–395. 21321607 10.1038/cr.2011.22 PMC3193420

[bib6] Filippakopoulos P, Knapp S. Targeting bromodomains: epigenetic readers of lysine acetylation. Nat Rev Drug Discov 2014; 13: 337–356. 24751816 10.1038/nrd4286

[bib7] Furey TS. ChIP-seq and beyond: new and improved methodologies to detect and characterize protein-DNA interactions. Nat Rev Genet 2012; 13: 840–852. 23090257 10.1038/nrg3306 PMC3591838

[bib8] Jenuwein T, Allis CD. Translating the histone code. Science 2001; 293: 1074–1080. 11498575 10.1126/science.1063127

[bib9] Seligson DB, Horvath S, Shi T, Yu H, Tze S, Grunstein M et al. Global histone modification patterns predict risk of prostate cancer recurrence. Nature 2005; 435: 1262–1266. 15988529 10.1038/nature03672

[bib10] Lizcano F, Garcia J. Epigenetic control and cancer: the potential of histone demethylases as therapeutic targets. Pharmaceuticals 2012; 5: 963–990. 24280700 10.3390/ph5090963 PMC3816642

[bib11] Chen HP, Zhao YT, Zhao TC. Histone deacetylases and mechanisms of regulation of gene expression. Crit Rev Oncog 2015; 20: 35–47. 25746103 10.1615/critrevoncog.2015012997 PMC4809735

[bib12] Venkatasubramani A V, McLaughlin K, Blanco G-R, Larionov V, Kagansky A. Pilot RNAi screening using mammalian cell-based system identifies novel putative silencing factors including Kat5/Tip60. AIMS Biophys 2015; 2: 570–584.

[bib13] Fraga MF, Ballestar E, Villar-Garea A, Boix-Chornet M, Espada J, Schotta G et al. Loss of acetylation at Lys16 and trimethylation at Lys20 of histone H4 is a common hallmark of human cancer. Nat Genet 2005; 37: 391–400. 15765097 10.1038/ng1531

[bib14] West AC, Johnstone RW. New and emerging HDAC inhibitors for cancer treatment. J Clin Invest 2014; 124: 30–39. 24382387 10.1172/JCI69738 PMC3871231

[bib15] Ma N, Luo Y, Wang Y, Liao C, Ye W-C, Jiang S. Selective histone deacetylase inhibitors with anticancer activity. Curr Top Med Chem 2016; 16: 415–426. 26268343 10.2174/1568026615666150813145629

[bib16] Bojang P, Ramos KS. The promise and failures of epigenetic therapies for cancer treatment. Cancer Treat Rev 2014; 40: 153–169. 23831234 10.1016/j.ctrv.2013.05.009 PMC3823804

[bib17] Kurdistani SK. Histone modifications in cancer biology and prognosis. Prog Drug Res 2011; 67: 91–106. 21141726 10.1007/978-3-7643-8989-5_5

[bib18] Donohoe DR, Bultman SJ. Metaboloepigenetics: interrelationships between energy metabolism and epigenetic control of gene expression. J Cell Physiol 2012; 227: 3169–3177. 22261928 10.1002/jcp.24054 PMC3338882

[bib19] Zhdanov R, Schirmer EC, Venkatasubramani A V, Kerr ARW, Mandrou E, Rodriguez-blanco G et al. Lipids contribute to epigenetic control via chromatin structure and functions. SciecnceOpen Res 2016; 10: 1–12.

[bib20] Akbani R, Becker K-F, Carragher N, Goldstein T, de Koning L, Korf U et al. Realizing the promise of reverse phase protein arrays for clinical, translational, and basic research: a workshop report: the RPPA (Reverse Phase Protein Array) society. Mol Cell Proteomics 2014; 13: 1625–1643. 24777629 10.1074/mcp.O113.034918 PMC4083105

[bib21] Kimura H, Hayashi-Takanaka Y, Goto Y, Takizawa N, Nozaki N. The organization of histone H3 modifications as revealed by a panel of specific monoclonal antibodies. Cell Struct Funct 2008; 33: 61–73. 18227620 10.1247/csf.07035

[bib22] Hayashi-Takanaka Y, Maehara K, Harada A, Umehara T, Yokoyama S, Obuse C et al. Distribution of histone H4 modifications as revealed by a panel of specific monoclonal antibodies. Chromosome Res 2015; 23: 753–766. 26343042 10.1007/s10577-015-9486-4 PMC4666908

[bib23] Latham T, MacKay L, Sproul D, Karim M, Culley J, Harrison DJ et al. Lactate, a product of glycolytic metabolism, inhibits histone deacetylase activity and promotes changes in gene expression. Nucleic Acids Res 2012; 40: 4794–4803. 22323521 10.1093/nar/gks066 PMC3367171

[bib24] Poleshko A, Kossenkov A V, Shalginskikh N, Pecherskaya A, Einarson MB, Skalka AM et al. Human factors and pathways essential for mediating epigenetic gene silencing. Epigenetics 2014; 9: 1280–1289. 25147916 10.4161/epi.32088 PMC4169020

[bib25] Tampakis A, Tampaki EC, Nebiker CA, Kouraklis G. Histone deacetylase inhibitors and colorectal cancer: what is new? Anticancer Agents Med Chem 2014; 14: 1220–1227. 25246306 10.2174/1871520614666140919095828

[bib26] Madiraju P, Pande S V, Prentki M, Madiraju SRM. Mitochondrial acetylcarnitine provides acetyl groups for nuclear histone acetylation. Epigenetics 2009; 4: 399–403. 19755853 10.4161/epi.4.6.9767

[bib27] Ferlay J, Soerjomataram I, Dikshit R, Eser S, Mathers C, Rebelo M et al. Cancer incidence and mortality worldwide: sources, methods and major patterns in GLOBOCAN. 2012Int J Cancer 2015; 136: E359–E386. 25220842 10.1002/ijc.29210

[bib28] Saltz LB, Cox J V, Blanke C, Rosen LS, Fehrenbacher L, Moore MJ et al. Irinotecan plus Fluorouracil and Leucovorin for metastatic colorectal cancer. N Engl J Med 2000; 343: 905–914. 11006366 10.1056/NEJM200009283431302

[bib29] de Gramont A, Figer A, Seymour M, Homerin M, Hmissi A, Cassidy J et al. Leucovorin and Fluorouracil with or without Oxaliplatin as first-line treatment in advanced colorectal cancer. J Clin Oncol 2000; 18: 2938–2947. 10944126 10.1200/JCO.2000.18.16.2938

[bib30] Weichert W, Röske A, Niesporek S, Noske A, Buckendahl AC, Dietel M et al. Class I histone deacetylase expression has independent prognostic impact in human colorectal cancer: Specific role of class I histone deacetylases in vitro and in vivo. Clin Cancer Res 2008; 14: 1669–1677. 18347167 10.1158/1078-0432.CCR-07-0990

[bib31] Duldulao MP, Lee W, Le M, Chen Z, Li W, Wang J et al. Gene expression variations in microsatellite stable and unstable colon cancer cells. J Surg Res 2012; 174: 1–6. 21816436 10.1016/j.jss.2011.06.016 PMC3210903

[bib32] Folkvord S, Ree AH, Furre T, Halvorsen T, Flatmark K. Radiosensitization by SAHA in experimental colorectal carcinoma models-in vivo effects and relevance of histone acetylation status. Int J Radiat Oncol Biol Phys 2009; 74: 546–552. 19427556 10.1016/j.ijrobp.2009.01.068

[bib33] Mwakwari SC, Patil V, Guerrant W, Oyelere AK. Macrocyclic histone deacetylase inhibitors. Curr Top Med Chem 2010; 10: 1423–1440. 20536416 10.2174/156802610792232079 PMC3144151

[bib34] Miller YE, Drabkin H, Jones C, Fisher JH. Human aminoacylase-1: cloning, regional assignment to distal chromosome 3p21.1, and identification of a cross-hybridizing sequence on chromosome 18. Genomics 1990; 8: 149–154. 1707030 10.1016/0888-7543(90)90237-o

[bib35] Ulanovskaya OA, Zuhl AM, Benjamin CF. NNMT promotes epigenetic remodelling in cancer by creating a metabolic methylation sink. Nat Chem Biol 2013; 9: 300–306. 23455543 10.1038/nchembio.1204 PMC3631284

[bib36] Bloch K, Rittenberg D. The metabolism of acetylamino acids. J Biol Chem 1947; 169: 467–476. 20259079

[bib37] Wagner JM, Hackanson B, Lubbert M, Jung M. Histone deacetylase (HDAC) inhibitors in recent clinical trials for cancer therapy. Clin Epigenetics 2010; 1: 117–136. 21258646 10.1007/s13148-010-0012-4 PMC3020651

[bib38] Bremer J. Carnitine—metabolism and functions. Physiol Rev 1983; 63: 1420–1480. 6361812 10.1152/physrev.1983.63.4.1420

[bib39] Li S, Li Q, Li Y, Li L, Tian H, Sun X. Acetyl-L-carnitine in the treatment of peripheral neuropathic pain: a systematic review and meta-analysis of randomized controlled trials. PLoS ONE 2015; 10: e0119479. 25751285 10.1371/journal.pone.0119479 PMC4353712

[bib40] Malaguarnera M, Gargante MP, Cristaldi E, Colonna V, Messano M, Koverech A et al. Acetyl L-carnitine (ALC) treatment in elderly patients with fatigue. Arch Gerontol Geriatr 2008; 46: 181–190. 17658628 10.1016/j.archger.2007.03.012

[bib41] Lu Y, Li N, Gao L, Xu Y-J, Huang C, Yu K et al. Acetylcarnitine is a candidate diagnostic and prognostic biomarker of hepatocellular carcinoma. Cancer Res 2016; 76: 2912–2920. 26976432 10.1158/0008-5472.CAN-15-3199

[bib42] Elimrani I, Dionne S, Saragosti D, Qureshi I, Levy E, Delvin E et al. Acetylcarnitine potentiates the anticarcinogenic effects of butyrate on SW480 colon cancer cells. Int J Oncol 2015; 47: 755–763. 26043725 10.3892/ijo.2015.3029

[bib43] Ruemmele FM, Dionne S, Qureshi I, Sarma DS, Levy E, Seidman EG. Butyrate mediates Caco-2 cell apoptosis via up-regulation of pro-apoptotic BAK and inducing caspase-3 mediated cleavage of poly-(ADP-ribose) polymerase (PARP). Cell Death Differ 1999; 6: 729–735. 10467346 10.1038/sj.cdd.4400545

[bib44] Roy M-J, Dionne S, Marx G, Qureshi I, Sarma D, Levy E et al. In vitro studies on the inhibition of colon cancer by butyrate and carnitine. Nutrition 2009; 25: 1193–1201. 19619983 10.1016/j.nut.2009.04.008

[bib45] Poleshko A, Einarson MB, Shalginskikh N, Zhang R, Adams PD, Skalka AM et al. Identification of a functional network of human epigenetic silencing factors. J Biol Chem 2010; 285: 422–433. 19880521 10.1074/jbc.M109.064667 PMC2804189

[bib46] Carpenter AE, Jones TR, Lamprecht MR, Clarke C, Kang IH, Friman O et al. CellProfiler: image analysis software for identifying and quantifying cell phenotypes. Genome Biol 2006; 7: R100. 17076895 10.1186/gb-2006-7-10-r100 PMC1794559

