# Supplementary material for: Global histone modification fingerprinting in human cells using epigenetic reverse phase protein array
Source: Cell Death Discov. 2017 Mar 6;3:16077–. doi: 10.1038/cddiscovery.2016.77 (PMC5349387; doi:10.1038/cddiscovery.2016.77)
Supplement: Supplementary Informatio [file cddiscovery201677-s1.doc]

**Supplementary Figures Description**

S1a : 96-well plate layout explained in the Materials and Methods (1^st^ dilution)

S1b, S1c, S1d : 384-well plate layouts explained in the Materials and Methods (2^nd^ dilution)

S2. : The effect of HDAC inhibitor HC-Toxin on expression of Acy1: HCT116 cells were treated with 100nM HC Toxin (black bars) for indicated time intervals (X axis) compared to DMSO control treatments (white bars). Acy1 transcript expression change, measured using qRT-PCR (described in Materials and Methods) relative to 0 time point was plotted (Y axis); 2 biological replicates with 3 technical replicates each time.
