# Supplementary figures and images for: Global histone modification fingerprinting in human cells using epigenetic reverse phase protein array
Source: Cell Death Discov. 2017 Mar 6;3:16077–. doi: 10.1038/cddiscovery.2016.77 (PMC5349387; doi:10.1038/cddiscovery.2016.77)

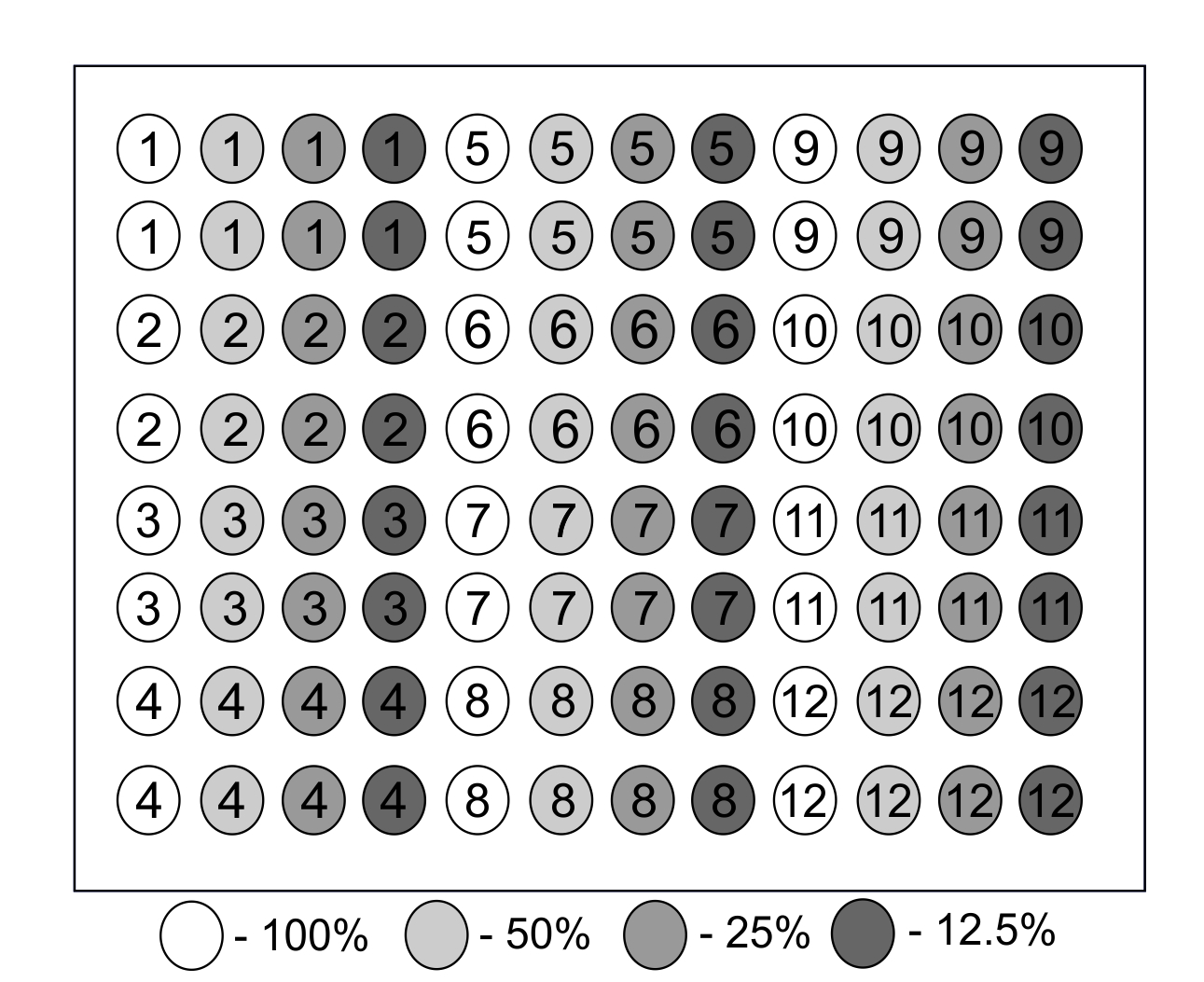

Supplement: Supplementary Informatio [file cddiscovery201677-s2.jpg]

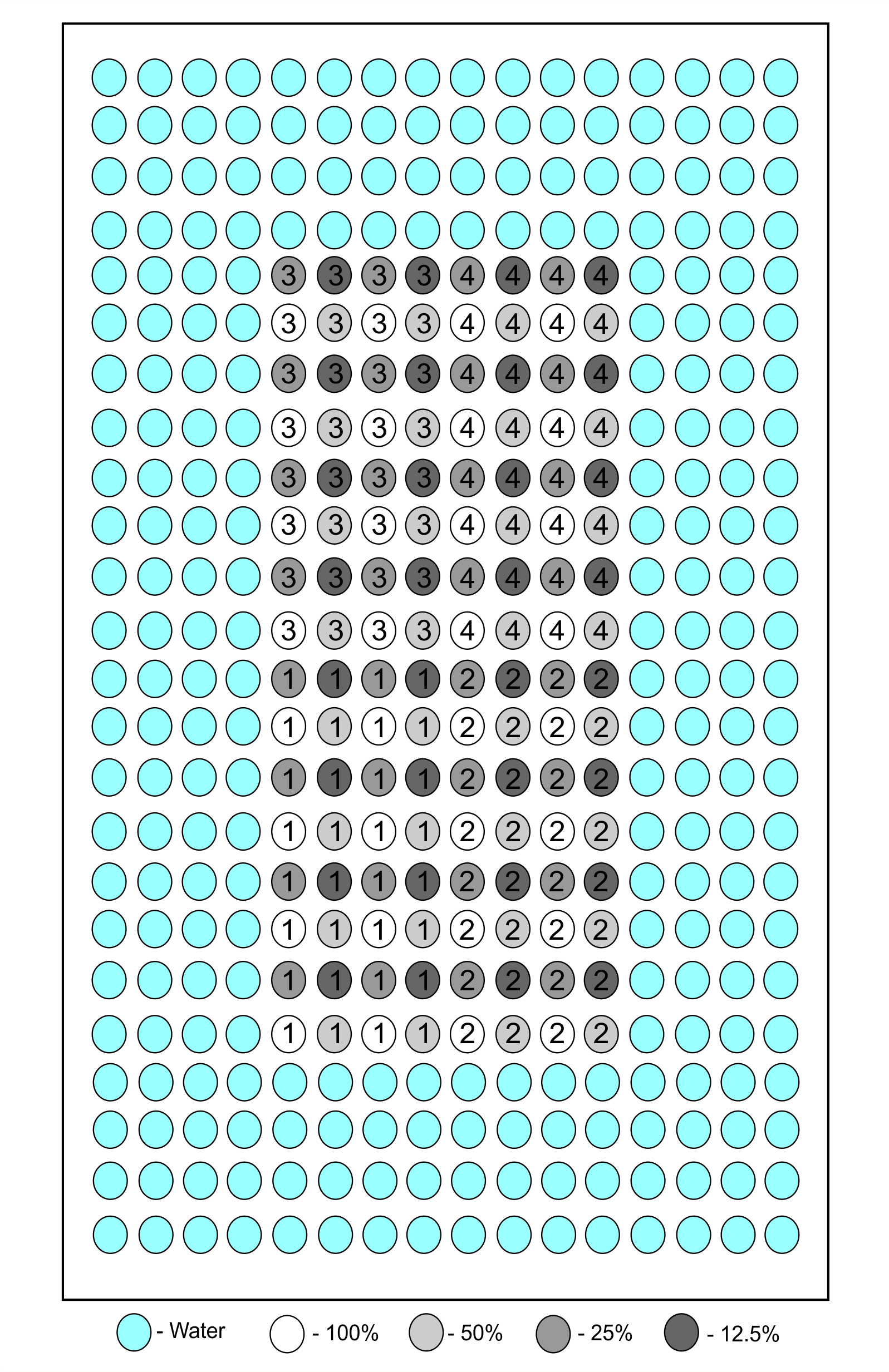

Supplement: Supplementary Informatio [file cddiscovery201677-s3.jpg]

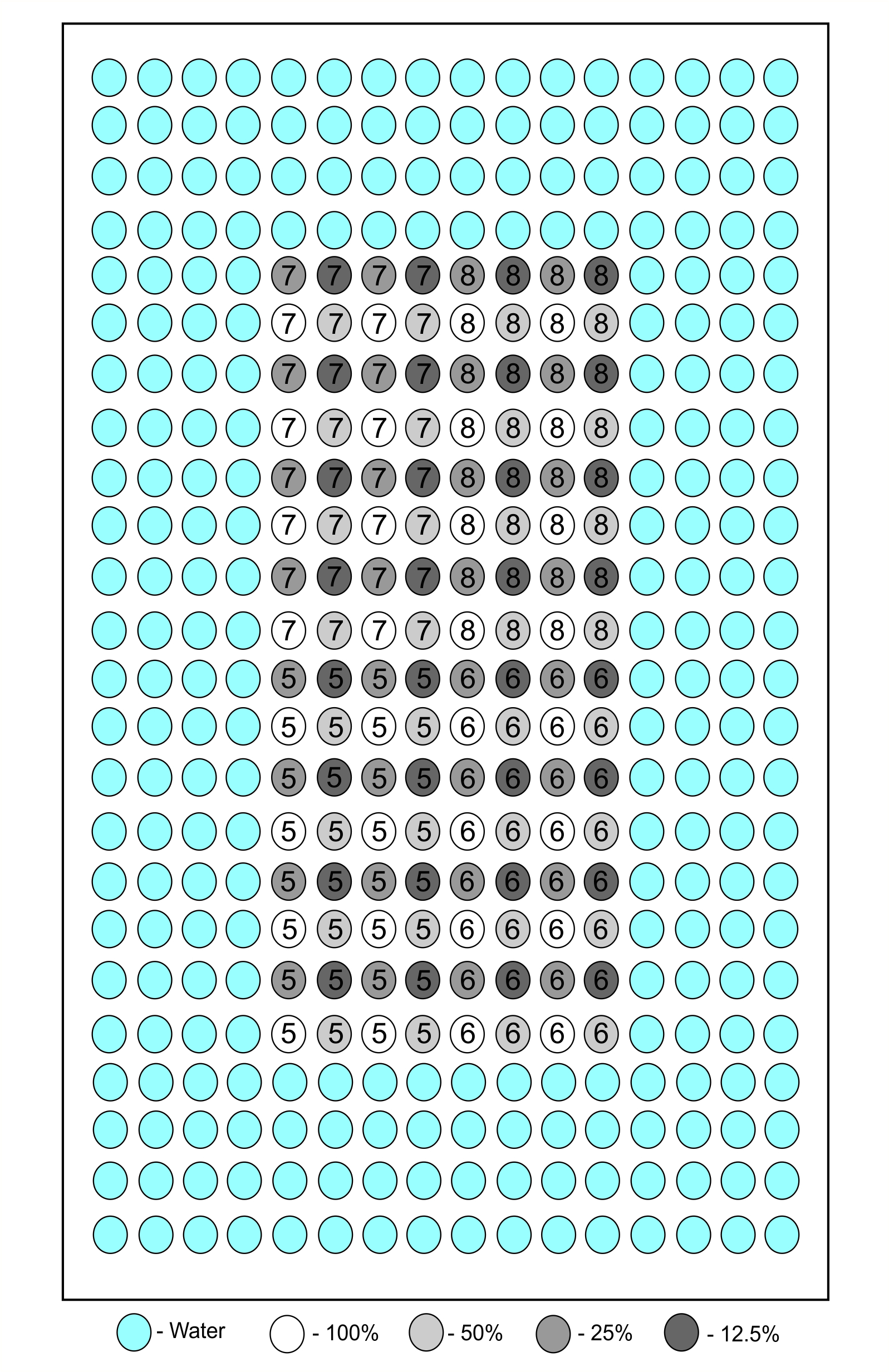

Supplement: Supplementary Informatio [file cddiscovery201677-s4.jpg]

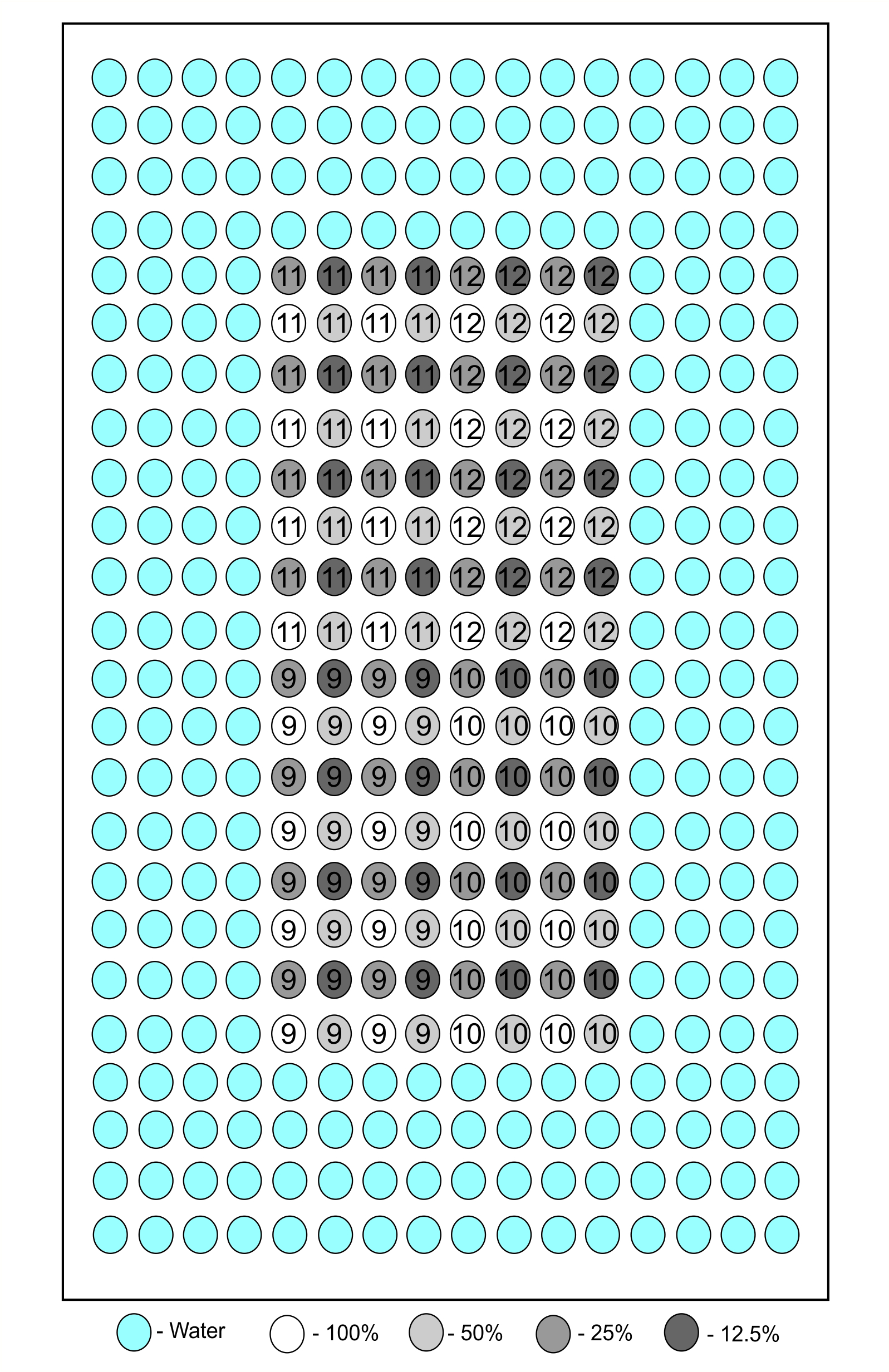

Supplement: Supplementary Informatio [file cddiscovery201677-s5.jpg]

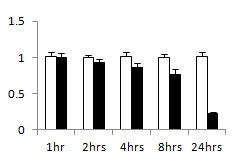

Supplement: Supplementary Informatio [file cddiscovery201677-s6.jpg]

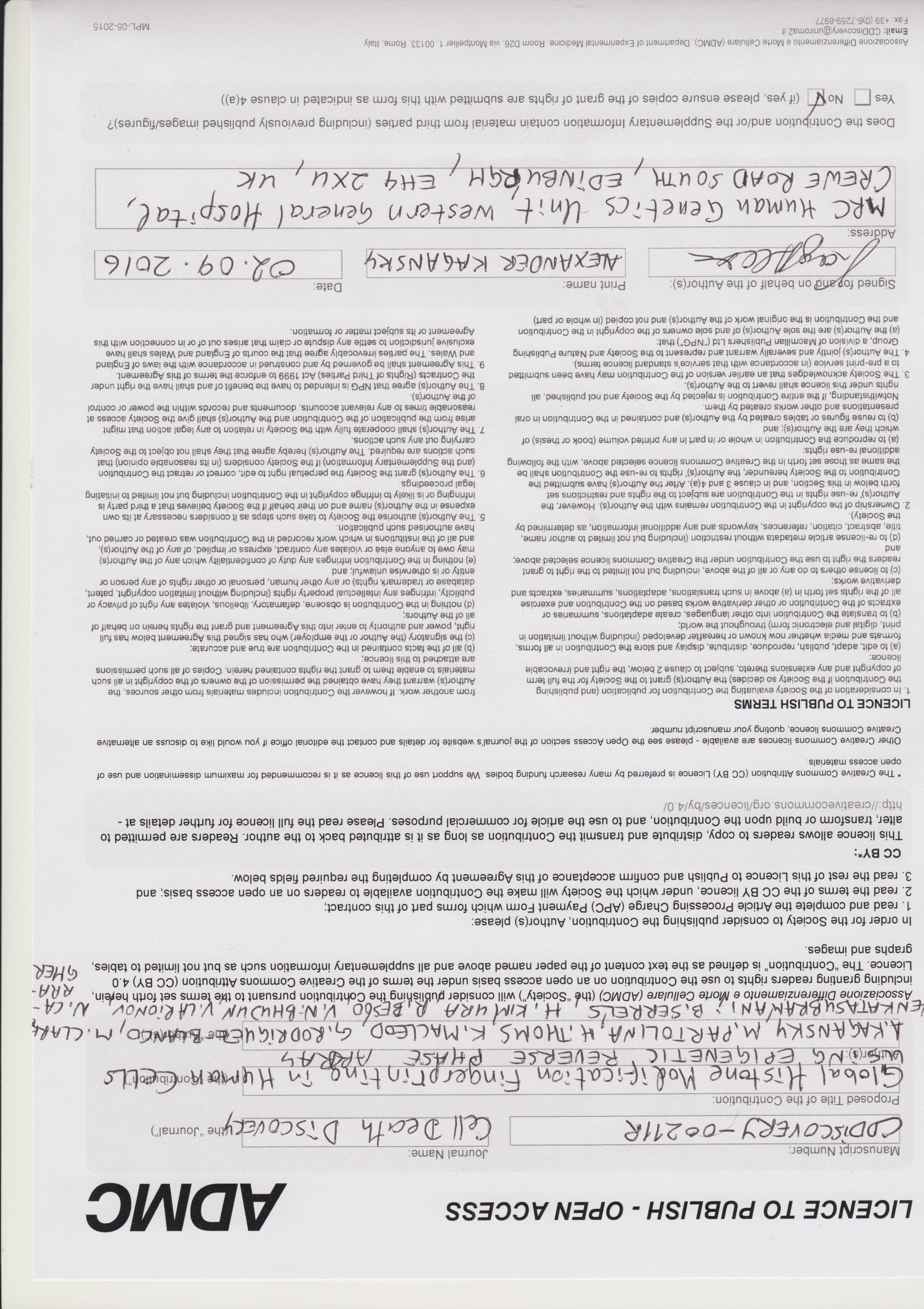

Supplement: Supplementary Informatio [file cddiscovery201677-s7.jpg]

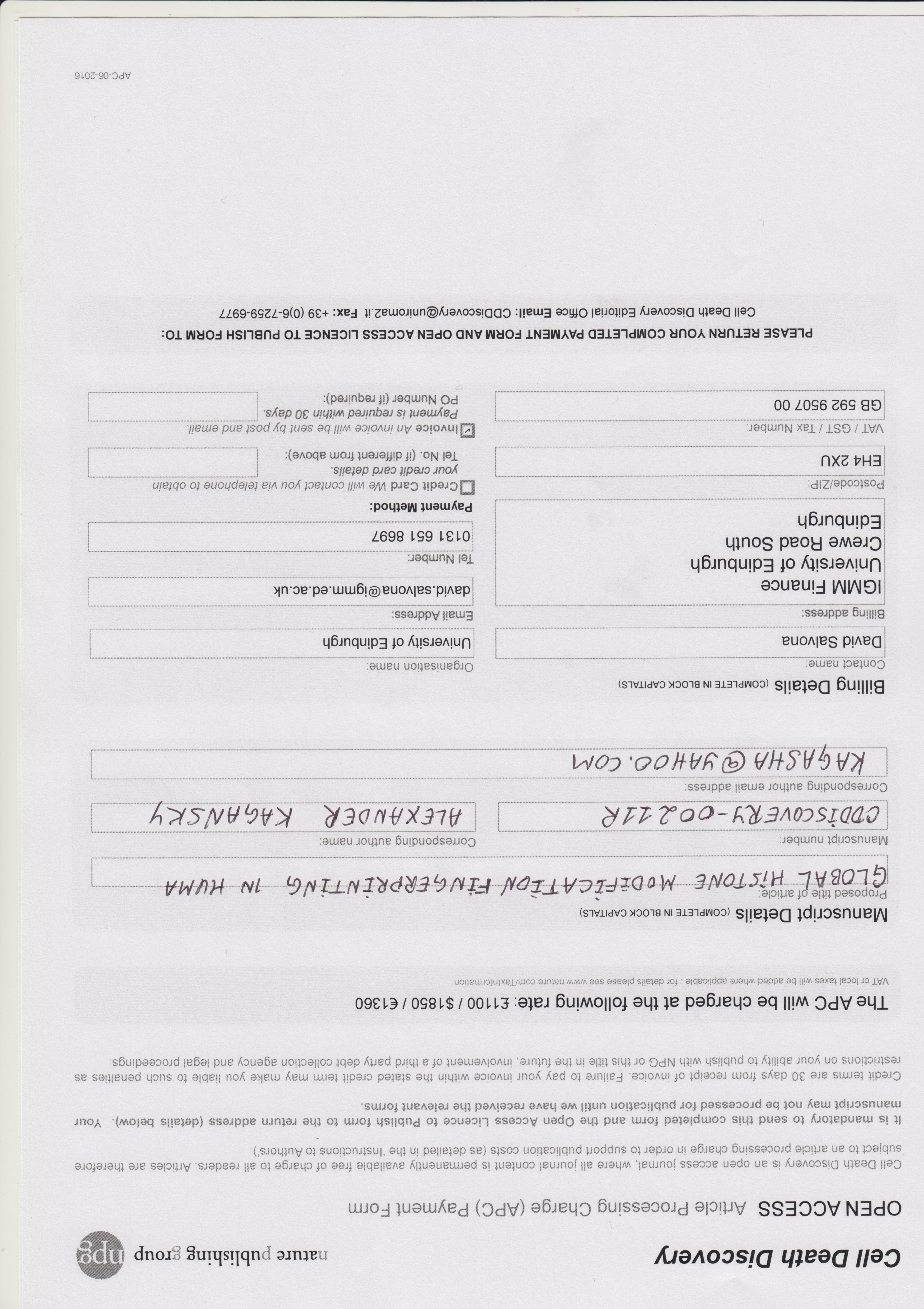

Supplement: Supplementary Informatio [file cddiscovery201677-s8.jpg]
